# Wernicke's Encephalopathy Following 40 Days of Water‐Only Fasting: A Case Report

**DOI:** 10.1002/ccr3.71049

**Published:** 2025-10-22

**Authors:** Ruba Adel Aweer, Noor A. Aweer, Omar Tluli, Anwar Joudeh

**Affiliations:** ^1^ College of Medicine QU Health, Qatar University Doha Qatar; ^2^ Department of Internal Medicine Al Khor Hospital, Hamad Medical Corporation Doha Qatar; ^3^ Internal Medicine, College of Medicine University of Qatar Doha Qatar

**Keywords:** fasting, nonalcoholic Wernicke encephalopathy, refeeding syndrome, thiamine deficiency, Wernicke encephalopathy

## Abstract

Wernicke's encephalopathy (WE) is an acute neurological disorder that develops due to thiamine (vitamin B1) deficiency. While it is common in chronic alcoholism, WE can also manifest due to other conditions, including extreme fasting. This report presents a unique case of WE occurring after a 40‐day water‐only fast, emphasizing the importance of recognizing nonalcoholic causes of WE. A 36‐year‐old male presented with diplopia, gait instability, and confusion following a 40‐day water‐only fast. Neurological examination revealed bilateral sixth cranial nerve palsy, ataxic gait, and confusion. Magnetic resonance imaging demonstrated bilateral medial thalami and periaqueductal hyperintensities, consistent with WE. He received high‐dose intravenous thiamine and electrolyte correction, resulting in substantial improvement. This case underscores the potential occurrence of WE in healthy individuals following extreme fasting. Early identification and treatment with thiamine are essential to prevent irreversible neurological damage. Clinicians should be cautious of WE in at‐risk patients without a history of alcohol use.

**Summary**

- Wernicke's encephalopathy (WE) can occur in healthy individuals following extreme fasting, not just in chronic alcohol users.
- Early recognition and high‐dose thiamine administration are crucial to prevent irreversible neurological damage.
- Clinicians should consider WE in patients with neurological symptoms following prolonged fasting, even without a history of alcohol use.

## 1 Introduction

Wernicke's encephalopathy (WE) is a critical neurological disorder resulting from thiamine (vitamin B1) deficiency [1]. Thiamine, when phosphorylated, plays a pivotal role as a coenzyme in numerous biochemical pathways within the brain. The body's thiamine reserves are sufficient for only 18 days without adequate intake [2]. Consequently, prolonged thiamine deficiency leads to brain lesions, particularly in regions of high thiamine content. Clinically, patients with WE exhibit diverse symptoms, making early detection challenging. However, the classic triad of WE includes ataxia, nystagmus, and mental status alterations. WE is most associated with chronic alcohol abuse. However, it may manifest due to nonalcoholic causes, such as malnutrition, hyperemesis gravidarum, malignancies, bariatric surgery, and gastrointestinal disorders [1, 3].

Wernicke's encephalopathy complicating water‐only fasting was rarely documented. To date, only two cases have been reported in the literature, making this case a significant addition [4, 5]. It emphasizes that WE can develop in otherwise healthy individuals with no history of recent alcohol intake who undergo extreme caloric restrictions and prolonged fasting. Nonalcoholic causes of WE should not be overlooked, particularly cases related to malnutrition and extreme fasting, as these practices are common in certain religious beliefs.

## 2 Case Report

### 2.1 Case History and Examination

A 36‐year‐old male patient presented to the emergency department with persistent double vision and gait instability that had persisted for one week. His companion observed that the patient appeared mildly confused and frequently experienced forgetfulness. These symptoms manifested two days prior to the patient's presentation, coinciding with the completion of a 40‐day religious fast during which he abstained from food and supplements, consuming only water. The patient denied headaches, dizziness, numbness, difficulty swallowing, fever, loss of consciousness, seizures, or a prior history of head or eye trauma. He reported a subjective reduction in urine output but no other gastrointestinal, respiratory, cardiovascular, or genitourinary symptoms. His medical, surgical, and pharmacological history was unremarkable. He had a previous history of excessive alcohol consumption and smoking, which he indicated he had ceased two and three years ago, respectively. The patient's vital signs were normal. Neurological exam showed disorientation, confusion, bitemporal hemianopia, bilateral 6th cranial nerve palsy, and facial twitching. He had difficulty following commands and an ataxic gait. Muscle tone, reflexes, and sensory exam were normal. Systemic exam was unremarkable.

### 2.2 Differential Diagnosis, Investigations and Treatment

**FIGURE 1:**
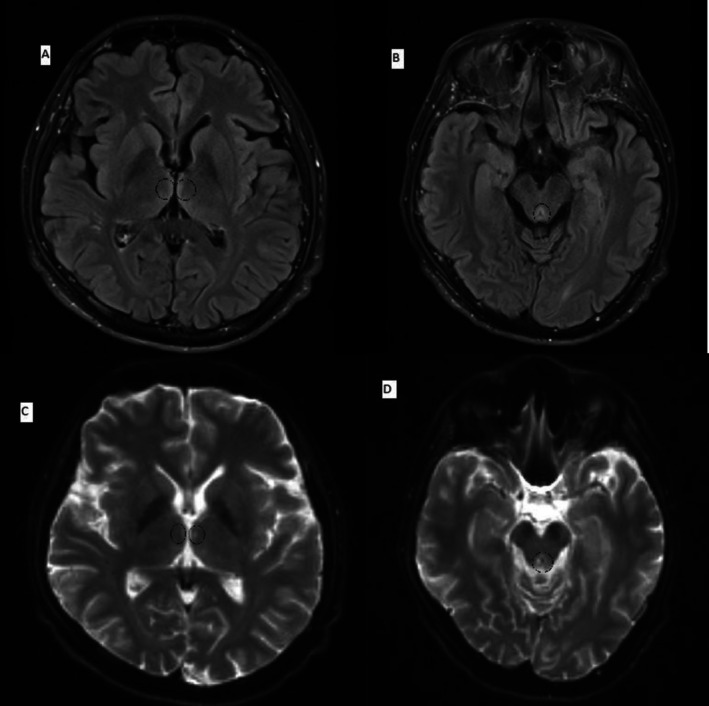
MRI of the brain demonstrating hyperintense lesions in the bilateral medial thalami (A) and periaqueductal region (B). These areas exhibit mild diffusion restriction on the corresponding diffusion‐weighted images (DWI) (C, D).

Baseline laboratory profiles indicated mild normocytic anemia (hemoglobin 12.3 g/dL [13–17 g/dL]), hypoalbuminemia (serum albumin 32 g/L [35–50 g/L]), and mild metabolic alkalosis (HCO_3_ 31 mmol/L [22–29 mmol/L]). Initial head Computed Tomography (CT) scans revealed an incidental venous developmental anomaly in the right parietal lobe. However, a subsequent magnetic resonance imaging (MRI) of the brain revealed T2 hyperintensities in the bilateral medial thalami and periaqueductal region, accompanied by mild diffusion restriction (Figure 1). These imaging findings, coupled with the laboratory results and clinical manifestations, confirmed the diagnosis of WE.

The patient received intravenous (IV) thiamine administration at a high dose of 500 mg thrice daily for three days, followed by 250 mg once daily for five days. Following admission, the patient developed electrolyte disturbances, specifically hypophosphatemia (0.25 mmol/L [0.8–1.5 mmol/L]), hypokalemia (2.8 mmol/L [3.5–5.3 mmol/L]), and hypomagnesemia (0.69 mmol/dL [0.7–1.0]), consistent with refeeding syndrome (Table 1). These were managed through IV electrolyte replacement. Caloric intake was subsequently restricted to 800 kcal/day and gradually increased to 1000 kcal/day under close supervision. The patient's dietary intake and electrolyte levels were closely monitored and regulated. Rehabilitation and occupational therapy were commenced for the patient.

**TABLE 1:** Summary of laboratory findings during hospitalization.

Date	Phosphate (mmol/L)	Magnesium (mmol/L)	Potassium (mmol/L)	Sodium (mmol/L)	Albumin (g/L)	Vitamin B1 (nmol/L)	Vitamin B12 (pmol/L)
16.02	0.81 ↓	0.66 ↓	3.8	137	43	472	583
17.02	0.91	0.86	3.8	137	38	—	583
18.02	0.72 ↓	0.69 ↓	3.7	142	35	—	—
19.02	1.25	0.74	2.8 ↓↓	142	32 ↓	—	—
20.02	1.58	0.83	4.4	137	34 ↓	—	—
21.02	1.48	0.89	4.1	141	34 ↓	—	—
23.02	1.34	0.84	4.0	138	32 ↓		268
25.02	1.46	0.84	3.7	139	27 ↓	> 472	

*Note:* ↓ Low; ↓↓ critically low.

### 2.3 Outcome and Follow‐Up

Over the subsequent days, the patient's condition exhibited significant improvement. Diplopia and gait instability gradually resolved, and he regained the ability to ambulate independently. Eight days after IV thiamine administration, his neurological status stabilized, facilitating the transition to oral thiamine and multivitamins. Additionally, he received a vitamin D injection of 300,000 units intramuscularly. Electrolyte levels remained stable with continued dietary adjustments and monitoring.

Upon discharge, the patient demonstrated the ability to ambulate independently with enhanced balance and complete resolution of diplopia. Discharge medications included daily oral multivitamin and vitamin B complex tablets. Dietary instructions emphasized the gradual progression of nutrient‐rich foods in accordance with dietitian recommendations, along with strict avoidance of prolonged fasting or inadequate nutritional intake.

One week after discharge, he was still having mild ataxia and fatigue. Ophthalmological examination revealed partial resolution of diplopia, with persistent left sided hazy vision, and left gaze end point nystagmus on ocular motility testing. Three weeks later, he was still complaining of dizziness, but his vision and ataxia improved. Lastly, the patient became asymptomatic at six‐week follow up.

## 3 Discussion

The presented case elucidates a rare yet significant manifestation of WE following a 40‐day water‐only fast. The paucity of documented cases in healthy individuals underscores a knowledge gap, considering the prevalent religious and cultural fasting practices. This case contributes substantially to the extant literature on nonalcoholic WE by establishing prolonged fasting as the primary etiological factor in its development. The patient, a previously healthy, young, non‐alcohol‐consuming male without known comorbidities, minimizes potential confounding variables and established risk factors for WE. This report stands in contrast to previously published cases of nonalcoholic WE, which typically involve elderly patients with comorbidities, lymphoma, and pancreatic pseudocysts [4, 6, 7].

In this case, the patient exhibited the classical triad of WE (ataxia, ophthalmoplegia, and confusion). However, it is crucial to acknowledge that less than 20% of WE patients present with all three features. Consequently, incorporating neuroimaging to corroborate the diagnosis is particularly beneficial in the absence of typical WE characteristics [1]. The patient's clinical presentation of WE was corroborated by a brain MRI scan demonstrating symmetric hyperintensities of the periaqueductal gray and dorsomedial thalami, which are thiamine‐sensitive brain regions. These findings can be attributed to the fact that thiamine deficiency impairs neuronal cellular energy production, glucose metabolism, and glutamate clearance, leading to excitotoxicity, neuroinflammation, and neuronal death [8]. If left untreated, this condition could have resulted in irreversible neurological damage, potentially underdiagnosed. Most critically, the patient's rapid clinical improvement following thiamine administration highlights the importance of initiating therapy in suspected WE cases, even before imaging confirmation, to prevent irreversible neurological complications. This case reinforces that timely treatment based on clinical suspicion is significant, as delayed interventions worsen the outcomes.

The multidisciplinary management plan employed in this case further enhances the approach. It effectively addressed both the acute neurological symptoms and the electrolyte abnormalities resulting from refeeding syndrome. Clinical evidence suggests that early administration of high‐dose IV thiamine is crucial in preventing irreversible neurological damage and optimizing recovery [1]. Refeeding syndrome, caused by the sudden influx of glucose and the redistribution of electrolytes into cells, is a potential complication of weaning treatment. Consequently, correction and close monitoring of electrolyte disturbances, particularly hypophosphatemia, hypokalemia, and hypomagnesemia, are paramount [9]. Our medical team commenced gradual refeeding of the patient as prescribed in the guidelines to mitigate life‐threatening complications such as arrhythmias, respiratory failure, and seizures associated with refeeding syndrome. A dietician was consulted to optimize the patient's intake and provide nutritional education. Furthermore, supportive measures were implemented, including venous thromboembolism prophylaxis, constipation management, alternate eye patching for diplopia, and the initiation of physical and occupational therapy to address lower limb weakness and ataxia.

However, this case report presents several limitations. Notably, there is no established diagnostic criterion for nonalcoholic Wernicke‐Korsakoff encephalopathy. Although the Caine criteria, which demonstrated greater sensitivity compared to the clinical WE triad, were proposed for diagnosing alcohol‐related WE, there are no such criteria in the current guidelines for nonalcoholic WE cases [10]. This absence of guidance can lead to diagnostic delays and underscores the urgent need for updated criteria specifically designed for nonalcoholic WE cases [1]. Another limitation worth mentioning is the absence of follow‐up neuroimaging after treatment receipt. This can hinder our assessment of the reversibility of neurological damage caused by thiamine deficiency. Follow‐up CT or MRI scans could have provided objective evidence regarding neurological recovery, facilitated the distinction between reversible and permanent brain lesions, and contributed valuable insights into disease resolution in nonalcoholic WE. Additionally, the patient should have been followed up for neurorehabilitation, as patients may continue to experience residual cognitive and motor deficits despite treatment. Incorporating rehabilitation into management pathways may therefore optimize recovery and long‐term outcomes [11].

Nonalcoholic Wernicke's encephalopathy (WE) has been increasingly recognized as a distinct clinical entity, most commonly associated with conditions such as hyperemesis gravidarum, bariatric surgery, cancer, and prolonged starvation. Compared to alcohol‐related WE, nonalcoholic patients are typically younger, more often female, and more frequently present with the complete classical triad of encephalopathy, ocular findings, and ataxia. MRI appears to be more sensitive in detecting lesions in this group, often involving the thalami and periaqueductal region.

A recurring feature in the nonalcoholic literature is extreme weight loss and persistent vomiting as warning signs, with many patients diagnosed only after significant neurological deterioration. Delayed recognition is common, and inadequate treatment frequently results in chronic cognitive sequelae such as Korsakoff's syndrome. Importantly, prophylactic parenteral thiamine (500 mg three times daily) is recommended in any high‐risk patient with rapid weight loss or reduced nutritional intake, as oral supplementation is insufficient in the presence of vomiting or malabsorption [12, 13].

This case highlights several crucial lessons in the diagnosis and management of Wernicke‐Korsakoff encephalopathy (WE). Firstly, it is paramount to maintain a high level of suspicion for WE in at‐risk patients, even in the absence of a current or recent alcohol intake history. Restricted caloric intake alone can lead to thiamine deficiency. Secondly, only a small proportion of patients with WE exhibit the classic triad. Therefore, atypical presentations require caution and meticulous observation, complemented by sound clinical judgment. In such emergency cases, diagnostic confirmation may be secondary to prompt intervention. Additionally, a history of alcohol use followed by fasting should prompt consideration for prophylactic thiamine supplementation to prevent severe neurological complications, such as Korsakoff syndrome.

## 4 Patient Perspective

“When I first started experiencing my symptoms, I thought they were just due to dehydration from my 40‐day religious fast. Even as my symptoms worsened over the week, I pushed through, determined to complete the fast. By the time I finally arrived at the hospital, I was completely drained—physically exhausted and spiritually overwhelmed. I remember feeling a strange sense of acceptance, almost ready for death. I thought if it was my time, then so be it. When the doctors told me I had WE, I couldn't fully process it. I felt numb. I had heard of others who didn't survive the 40‐day fast, yet somehow, I did. I believe it was nothing short of a miracle.

After the diagnosis, my emotions were a mix of self‐pity and spiritual reassurance. Sometimes, I question my decision to undergo such an extreme fast. But then, I remind myself of my commitment to my faith. It's a struggle—balancing regret and belief. The illness has taken a toll on my daily life. I constantly feel fatigued, like I have “the energy of a grandpa.” I've become more socially withdrawn, struggling to keep up with conversations or enjoy time with my colleagues.

My experience with healthcare has been overwhelmingly positive. The medical team treated me with compassion and dedication. They took the time to explain my condition and the treatment plan. I understood it, but I prefer not to dwell on it too much—it sometimes conflicts with my spiritual beliefs. I know some might see my fasting as extreme. But I believe that my faith played a crucial role in my survival and recovery. To me, it was a test, and I made it through by grace.”

## Author Contributions

**Ruba Adel Aweer:** conceptualization, data curation, writing – original draft, writing – review and editing. **Noor A. Aweer:** conceptualization, data curation, writing – original draft, writing – review and editing. **Omar Tluli:** conceptualization, data curation, writing – original draft, writing – review and editing. **Anwar Joudeh:** conceptualization, supervision, writing – review and editing.

## Ethics Statement

This work was conducted in accordance with the Declaration of Helsinki (1964). This case report was approved by the Institutional Review Board at Hamad Medical Corporation, Doha, Qatar (reference number: MRC‐04‐25‐343).

## Consent

Written informed consent was obtained from the patient to publish this report in accordance with the journal's patient consent policy.

## Conflicts of Interest

The authors declare no conflicts of interest.

## Data Availability

Data can be obtained from the corresponding author upon request.
